# Apical surgery failures: Extraction or re-surgery? Report of five cases

**DOI:** 10.15171/joddd.2018.018

**Published:** 2018-06-20

**Authors:** Damla Torul, sevda kurt, Kamber Kamberoglu

**Affiliations:** ^1^Department of Oral and Maxillofacial Surgery, Faculty of Dentistry, Ondokuz Mayis University, Samsun, Turkey; ^2^Department of Periodontology, Recep Tayyip Erdoğan University, Rize, Turkey; ^3^Oral and Maxillofacial Surgeon, Hospitadent, Istanbul, Turkey

**Keywords:** Apicoectomy, extraction, retrograde obturation

## Abstract

Apical surgery (AS) is considered as the last attempt to save teeth which cannot be treated with conventional endodontic approach. The main goal of apical surgery is to create a barrier between the root-canal system and the peri-radicular tissues by means of a tight root-end filling after resection. However, failures in this treatment is usually result with tooth loss. In such cases surgical re-treatment would take into consideration as viable alternative. In this case series, successful ARs that performed in ten teeth of five patients who applied for extraction after an unsuccessful apical surgery, were presented. It is pointed that if appropriate surgical and endodontic intervention is performed and adequate apical obturation is provided with retrograde filling, teeth can be treat without extraction.

## Introduction

The aim of endodontic treatment is to eliminate microorganisms from the root canal system and create an effective barrier.^1,2^ Endodontic treatment may need to be renewed in cases of failure; however, when non-surgical treatment is inadequate, apical surgery (AS) is the only choice.^3-5^ AS is an endodontic surgical procedure which is performed by following steps such as root canal treatment, resection of the apical part of the root, preparation of adequate root-end filing and curettage of the periapical inflammation or necrotic tissue rigorously.^4^ In this surgical treatment, root-end filling plays a crucial role in providing an efficient apical obturation because the most common cause of failures in the apical surgery is inadequate obturation between the root canal system and peri-radicular tissues.^3,6^ In cases of apical surgery failure, apical re-surgery (AR) seems a viable alternative.^2,7^

It has also been reported that the use of guided tissue regeneration (GTR) has advantages such as promoting bone healing and increasing the success rate in AS; therefore, GTR and biomaterials can be applied in ASs.^1,3,5^

There are different opinions regarding AS and operative procedures in the literature. In this case group, ten cases which had previously undergone unsuccessful AS were treated with AR with different procedures, which are presented in light of the current literature.

## Case reports

**Figure 1:**
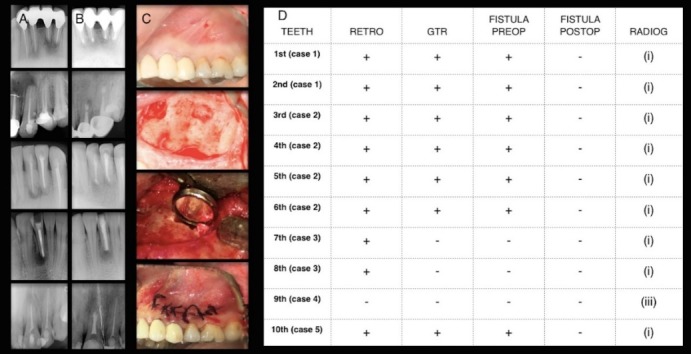
A. Preoperative radiographs; B. Postoperative radiographs (6^th^ month); C. Clinical photos of a case; D. Operative procedures, presence of fistula, radiographic healing

This report consists of five patients who had undergone unsuccessful AS in different health centers and applied to the Department of Oral and Maxillofacial Surgery of Ondokuz Mayis University from January 2016 to June 2017. Informed consent was obtained from the patients before inclusion. The patients were 20‒49 years of age. Three of the five patients were female and the remaining two were male. The systemic anamnesis of the patients was noncontributory. Re-surgery was planned and performed in all the patients by the same surgeon. Under local anesthesia with articaine hydrochloride (Ultracain D-S; Sanovi-Aventis, Istanbul, Turkey), a sulcular incision was performed and the bone cavity for each root was prepared using tungsten carbide fissure burs under copious saline irrigation. Surgical debridement of the bone was performed with an excavator rigorously. Because of the previous unsuccessful AS, the resection of the apex was performed in limited length with a bevel of 0‒10° and the borders of the previous preparation were corrected. An ultrasonic device (PM400; EMS, Nyon, Switzerland) was used at a medium power setting to prevent crack formation in dentin and to prepare approximately 3-mm root-end cavities. After isolation of the surgical area, IRM (Dentsply Caulk, Milford, DE, USA) root-end filing material was used to provide a tight apical seal in four of the patients. In one patient only, root canal treatment was renewed during the operation and root-end filling was not performed. A xenograft, Cerabone (Biotiss Biomaterial, Zossen, Germay), was used to fill the bone defects around the resected apex in three patients, while graft-less surgery was performed in other two patients (Figure 1). All the patients were followed for six months.

### Radiographic assessment

Radiographs were taken prior to the operation and after six months. From a practical point of view, small osteotomy procedures lead to healing of small (<5 mm) periapical defects in an average of six months.^8^ Therefore, the evaluation in our patients was performed at least six months after surgery.

Two endodontists, blinded to the cases and with at least five years of clinical experience, evaluated the radiographs. The preoperative radiograph and the final one were evaluated according to the classification of Rud et al^9^ (1972) as (i) complete healing; (ii) incomplete healing; (iii) uncertain healing; and (iv) unsatisfactory healing.

### Clinical evaluation

A routine examination of clinical signs and symptoms such as swelling, loss of function, tenderness to percussion or palpation, discomfort, mobility, sinus tract formation, or periodontal pocket formation was performed to identify and evaluate the prognosis on every recall visit (Table 1).

**Table 1:** Clinical outcomes prior to the operation and at 6th month recall visit

	**Swelling**		**TPP**		**Discomfort**		**Mobility**		**STF**		**PPF**	
												
	**B**	**6** ^th^ ** M**	**B**	**6** ^th^ ** M**	**B**	**6** ^th^ ** M**	**B**	**6** ^th^ ** M**	**B**	**6** ^th^ ** M**	**B**	**6** ^th^ ** M**
**1 (Case 1)**	**+**	**-**	**+**	**-**	**+**	**-**	**+**	**-**	**+**	**-**	**-**	**-**
**2 (Case 1)**	**+**	**-**	**+**	**-**	**+**	**-**	**+**	**-**	**+**	**-**	**-**	**-**
**3 (Case 2)**	**-**	**-**	**-**	**-**	**+**	**-**	**-**	**-**	**+**	**-**	**-**	**-**
**4 (Case 2)**	**-**	**-**	**-**	**-**	**+**	**-**	**-**	**-**	**+**	**-**	**-**	**-**
**5 (Case 2)**	**-**	**-**	**+**	**-**	**+**	**-**	**-**	**-**	**+**	**-**	**-**	**-**
**6 (Case 2)**	**-**	**-**	**+**	**-**	**+**	**-**	**-**	**-**	**+**	**-**	**-**	**-**
**7 (Case 3)**	**-**	**-**	**+**	**-**	**+**	**-**	**-**	**-**	**-**	**-**	**-**	**-**
**8 (Case 3)**	**-**	**-**	**+**	**-**	**+**	**-**	**-**	**-**	**-**	**-**	**-**	**-**
**9 (Case 4)**	**+**	**-**	**+**	**+**	**+**	**+**	**+**	**+**	**-**	**-**	**-**	**-**
**10 (Case 5)**	**+**	**-**	**+**	**-**	**+**	**-**	**-**	**-**	**+**	**-**	**-**	**-**

**B:** Baseline**; M:** Month**; TPP:** Tenderness to percussion or palpation; **STF:** Sinus track formation; **PPF:** Periodontal pocket formation

## Discussion

AS is considered as the last resort to preserve natural teeth after the failure of endodontic treatment.^10,11^ The main goal of AS is to create a tight seal in the root apex and thereby to prevent the occurrence of a pathway between the root canal system and peri-radicular tissues.^10,12,13^ With the use of modern surgical techniques and equipment, the reported success rates of this procedure has increased to almost 92%.^5,6^ However, failures still occur as a result of various reasons such as poor previous root canal treatment, inadequate resection of the root apex, absent or improperly prepared root-end cavity, inappropriate intra-surgical application of filling materials during the first surgery and inappropriate coronal restorations.^2,7,14^ Taschieri et al^6^ explored the reasons for the failure of endodontic surgery using scanning electron microscopy (SEM) and reported that the most common cause of failures include the absence of root-end filling and the presence of a gap between the root-end filling and dentin. Similarly, Song et al^2^ reported that absence of root-end filing and incorrect root-end preparations constitute the main causes of failures.

In case of AS failure, re-surgery might be an alternative approach, enabling clinicians to save the tooth after failure of the first surgery.^4^ In the literature, however, limited information is available regarding the success and clinical outcomes of AR. In a meta-analysis by Peterson and Gutmann^15^ (2001), the success of AR was found to be %36. This low success rate can be attributed to the use of old techniques and equipment in most of the studies included in the analysis. In 2005 Gagliani et al^7^ compared the success of the first and second surgeries in a five-year study and reported a 59% success rate in the re-surgery group compared with 86% in the teeth subjected to the first surgical procedure. Saunders^16^ performed a prospective outcome study of AR by using microsurgical techniques and MTA and reported a clinical success rate of 74.5% for re-surgery cases. In another study by Song et al^2^ (2011), all the surgical procedures were performed under an operating microscope with the use of MTA or Super EBA. Successful outcomes of this study were reported to be 92.9%. Although heterogeneities exist, it is clear that with the innovations in peri-radicular surgical approaches and materials used, AR may be considered a valid alternative.

.

In our series, AR of ten teeth was performed in five patients and failure was observed in only one tooth. The reasons for the failures of the first surgeries in our series were detected as poor previous root canal treatment, inadequate resection of the apex and mostly the absence of root-end filing. Christiansen et al^17^ reported that the success rate of treatment without a root-end filling was significantly low that was observed in teeth with root-end filling. Thus, it is a matter of fact that technically and biologically adequate management of the root-end is a perquisite for the success of apical surgery procedures.^3,6^ The commercial use of ultrasonic and sonic devices since 1990s simplified the preparation of root-end cavities and made it possible for clinicians to create a precise root-end cavity.^10-12^ In addition, regarding apical filling materials a wide variety of biocompatible materials have been introduced like MTA, EBA and IRM. MTA is the gold standard in apical sealing; however, it has been reported that EBA and IRM also yield results similar to those with the use of MTA.^4,11,18^ IRM is a zinc oxide‒eugenol cement, reinforced with polymethyl methacrylate and has been widely used because of its sealing ability and cost effectiveness, in apical surgeries.^19^ In 2003, Chong and Pitt Ford^20^ compared IRM and MTA with the use of similar microsurgical techniques. The results showed that both materials promoted high levels of healing (92% with MTA and 87% with IRM). We used IRM as an apical sealer successfully in nine patients. In only one patient there was failure after re-surgery. We think that this might be attributed to the absence of root-end sealing.

Regeneration of bone, periodontal ligament and cementum is required to a complete periapical healing after periapical surgery.^5,12^ It was reported that the prognosis was worsened by the loss of buccal bone plate after endodontic surgery.^21^ The use of GTR techniques has been proposed as an adjunct to endodontic surgery in order to promote bone healing. Studies indicated that the use of a combination of membrane barriers and other agents, such as bone graft materials, has been reported as a viable option to promote healing.^1,5^ Bernabé et al^3^ reported a successful case of peri-radicular surgery with a combination of MTA and GTR in 2013. We used GTR in six teeth of three patients and similarly favorable osseous healing was observed.

## Conclusion

Undoubtedly the preservation of natural teeth is the main goal in clinical dentistry and the revolutions in equipment/biomaterials enable clinicians to accomplish this goal. The hopeless teeth of the past can be treated with high success rates currently. This prevents patients, especially the young patients, from facing functional, esthetic and psychological shortcomings of tooth loss. In our series AR was performed with high success. We think that the key factor in the success of AR is provision of an efficient apical obturation in combination with rigorous curettage of necrotic tissues and AR is a potential alternative treatment modality in cases of AS failure.

## Author Contributions

DT, SK and KK were responsible for the concept of the study. KK carried out the surgeries. DT and SK carried out the literature review. DT, SK and KK prepared the methodology of the study. DT and SK prepared the original draft. DT, SK and KK reviewed and revised the manuscript.

## Acknowledgements

None.

## Funding

Not applicable.

## Conflict of Interests

The authors declare no competing interests with regards to the authorship and/or publication of this article.

## Ethical Approval

All the patients gave written consent for the publication of this paper.

## References

[1] Sanchez-Torres A, Sanchez-Garces MA, Gay-Escoda C (2014). Materials and prognostic factors of bone regeneration in periapical surgery: a systematic review. Med Oral Patol Oral Cir Bucal.

[2] Song M, Shin SJ, Kim E (2011). Outcomes of endodontic micro-resurgery: a prospective clinical study. J Endod.

[3] Bernabe PF, Azuma MM, Ferreira LL, Dezan-Junior E, Gomes-Filho JE, Cintra LT (2013). Root reconstructed with mineral trioxide aggregate and guided tissue regeneration in apical surgery: a 5-year follow-up. Braz Dent J.

[4] Tsurumachi T (2013). Current strategy for successful periradicular surgery. J Oral Sci.

[5] Tsesis I, Rosen E, Tamse A, Taschieri S, Del Fabbro M (2011). Effect of guided tissue regeneration on the outcome of surgical endodontic treatment: a systematic review and meta-analysis. J Endod.

[6] Taschieri S, Bettach R, Lolato A, Moneghini L, Fabbro MD (2011). Endodontic surgery failure: SEM analysis of root-end filling. J Oral Sci.

[7] Gagliani M, Taschieri S, Molinari R (1998). Ultrasonic root-end preparation: influence of cutting angle on the apical seal. J Endod.

[8] Rubinstein RA, Kim S (1999). Short-term observation of the results of endodontic surgery with the use of a surgical operation microscope and Super-EBA as root-end filling material. Journal of Endodontics.

[9] Rud J, Andreasen JO, Jensen JE (1972). Radiographic criteria for the assessment of healing after endodontic surgery. Int J Oral Surg.

[10] von Arx T, Jensen SS, Hanni S (2007). Clinical and radiographic assessment of various predictors for healing outcome 1 year after periapical surgery. J Endod.

[11] Kim S, Kratchman S (2006). Modern endodontic surgery concepts and practice: a review. J Endod.

[12] von Arx T, Kurt B (1999). Root-end cavity preparation after apicoectomy using a new type of sonic and diamond-surfaced retrotip: a 1-year follow-up study. J Oral Maxillofac Surg.

[13] Mobilio N, Vecchiatini R, Vasquez M, Calura G, Catapano S (2017 Summer). Effect of flap design and duration of surgery on acute postoperative symptoms and signs after extraction of lower third molars: A randomized prospective study. J Dent Res Dent Clin Dent Prospects.

[14] Lin LM, Skribner JE, Gaengler P (1992). Factors associated with endodontic treatment failures. J Endod.

[15] Peterson J, Gutmann JL (2001). The outcome of endodontic resurgery: a systematic review. Int Endod J.

[16] Saunders WP (2008). A prospective clinical study of periradicular surgery using mineral trioxide aggregate as a root-end filling. J Endod.

[17] Christiansen R, Kirkevang LL, Horsted-Bindslev P, Wenzel A (2009). Randomized clinical trial of root-end resection followed by root-end filling with mineral trioxide aggregate or smoothing of the orthograde gutta-percha root filling 1 year follow-up. Int Endod J.

[18] von Arx T (2011). Apical surgery: A review of current techniques and outcome. Saudi Dent J.

[19] Crooks WG, Anderson RW, Powell BJ, Kimbrough WF (1994). Longitudinal evaluation of the seal of IRM root end fillings. J Endod.

[20] Chong BS, Pitt Ford TR, Hudson MB (2009). A prospective clinical study of Mineral Trioxide Aggregate and IRM when used as root-end filling materials in endodontic surgery 2003. Int Endod J.

[21] Taschieri S, Del Fabbro M, Testori T, Weinstein R (2007). Efficacy of xenogeneic bone grafting with guided tissue regeneration in the management of bone defects after surgical endodontics. J Oral Maxillofac Surg.

